# Evaluation and Characterization of Ultrathin Poly(3-hydroxybutyrate) Fibers Loaded with Tetraphenylporphyrin and Its Complexes with Fe(III) and Sn(IV)

**DOI:** 10.3390/polym14030610

**Published:** 2022-02-04

**Authors:** Svetlana G. Karpova, Natalia A. Chumakova, Anton V. Lobanov, Anatoly A. Olkhov, Alexandre A. Vetcher, Alexey L. Iordanskii

**Affiliations:** 1 N.M. Emanuel Institute of Biochemical Physics, Russian Academy of Sciences, 4 Kosygin St., 119991 Moscow, Russia; karpova@sky.chph.ras.ru (S.G.K.); avlobanov@mail.ru (A.V.L.); aolkhov72@yandex.ru (A.A.O.); 2 Department of Chemistry, Lomonosov Moscow State University, 1 Kolmogorov St., 119991 Moscow, Russia; harmonic2011@yandex.ru; 3 N.N. Semenov Federal Research Center for Chemical Physics, Russian Academy of Sciences, 4 Kosygin St., 119334 Moscow, Russia; 4 Academic Department of Innovational Materials and Technologies Chemistry, Plekhanov Russian University of Economics, 36 Stremyanny Ln, 117997 Moscow, Russia; 5 Institute of Biochemical Technology and Nanotechnology (IBTN), Peoples’ Friendship University of Russia (RUDN), 6 Miklukho-Maklaya St., 117198 Moscow, Russia; 6 Complementary and Integrative Health Clinic of Dr. Shishonin, 5 Yasnogorskaya St., 117588 Moscow, Russia

**Keywords:** poly(3-hydroxybutyrate), biodegradable polyester, ultrafine electrospun fibers, tetraphenylporphyrin, metalloporphyrin complexes, Fe(III), Sn(IV), X-ray diffraction, DSC, spin probe EPR method, SEM

## Abstract

The effect of small additions (1–5 wt.%) of tetraphenylporphyrin (TPP) and its complexes with Fe (III) and Sn (IV) on the structure and properties of ultrathin fibers based on poly(3-hydroxybutyrate) (PHB) has been studied. A comprehensive study of biopolymer compositions included X-ray diffraction (XRD), differential scanning calorimetry (DSC), spin probe electron paramagnetic resonance method (EPR), and scanning electron microscopy (SEM). It was demonstrated that the addition of these dopants to the PHB fibers modifies their morphology, crystallinity and segmental dynamics in the amorphous regions. The annealing at 140 °C affects crystallinity and molecular mobility in the amorphous regions of the fibers, however the observed changes exhibit multidirectional behavior, depending on the type of porphyrin and its concentration in the fiber. Fibers exposure to an aqueous medium at 70 °C causes a nonlinear change in the enthalpy of melting and challenging nature of a change of the molecular dynamics.

## 1. Introduction

The widespread employment of nanotechnology in modern medicine, the transition from traditional macro- and micro- to submicron and nanoscale medication forms (MF), as well as the implementation of ultrafine implants and diagnostic systems, cause the scientific community to pay close attention to bio-based polymer materials that are completely decomposed in the living systems without the formation of toxic products. Poly(3-hydroxybutyrate) (PHB) as a basic representative of polyhydroxyalkanoates’ family (PHA) is such bacterial biodegradable and biocompatible polymer with great commercial perspectives and high sustainability [1,2,3]. PHB micro/nano fibers have been fabricated by electrospinning (ES), which is well-developed for creating nanoscale polymer carriers with adjustable morphology and properties [4,5] The ES technique provides the design of nonwoven fibrous membranes (mats) with a large inherent surface-to-volume ratio [6] that is extremely important for biomedical, packaging, and environmental applications.

The crucial characteristics of the biodegradable MF are the kinetic parameters of drug release in vitro or in vivo which are determined by the combination of drug diffusion and biopolymer decomposition rate in the limited space of fibrous micro- nanocarrier. Since the drug diffusional transport depends strongly on the inherent structure of the biopolymer, its morphology and crystallinity, all of these characteristics can significantly affect the kinetic profile of drug release, and, eventually, the effectiveness of the MF implementation. Structural changes in fibrillar mats during storage and can be caused by multiple factors, such as water absorption, heating, oxidation, ozonolysis, action of UV radiation, as well as the degradation effect of microorganisms. The listed factors can act simultaneously or consequently, depending on the operating conditions of the MF and the environment.

The effective method of direct impact on the structure of polymer materials is to dope it with the modifiers of organic and inorganic natures. In our preliminary reports, we demonstrated the influence of a series of additives on the structure of PHB-based fibrous materials. As the modifiers we used dipyridamole [7], chitosan [8], TiO_2_ and silicon nanoparticles [9], Fe-chloroporphyrin [10], Zn-porphyrin [11], Mg-chloroporphyrin [12], etc., that could be used as the therapeutic agents. The effect of low-molecular substances on the structure of the crystalline and amorphous phases of PHB fibers was shown in the above-mentioned publications. All these substances interact with the polyester groups of PHB. As a result of such interactions, both deceleration and acceleration of crystallinity, orientation, and relaxation of macromolecules have occurred. Obviously, for the formation of matrices with desired properties, it is necessary to establish the relationship between the structure of bioactive dopants and their impact on the structural and dynamic parameters of the fibrous material.

The challenges related to the variation of free volume [13,14] and structural organization [15,16] of the micro- and nanofibers stimulate molecular/segmental mobility investigations. The free volumes in polar fibrous polymers were found to be localized mainly at the chain ends [13], contributing to total free volume. However, for the biopolymers with poor polarity such as a highly crystalline PHB, the free volume should locate predominantly in intercrystalline amorphous areas where the transitive macromolecules are situated. The comprehensive exploration of drug delivery therapeutic systems on the base of polysorbate-80 and the cyclodextrin derivative has been performed to disclose the macro- and microstructure characterization in combination with free volume distribution study [15]. The innovative method of ortho-positronium annihilation was implemented there to display the free volume variation as response on intrinsic characteristics of drug delivery system [15]. The correlation among drug release, free volume concentration and segmental dynamics is a key factor of diffusion processes that control the drug release profiles in planar and fibrillar polymer systems [16,17].

Of particular interest is the incorporation of porphyrin metal complexes as the special dopants into the PHB polymer. It is well known from the numerous literature papers that the complexes of metals with TPPs have unique photocatalytic and antimicrobial properties [18,19]. Metalloporphyrin complexes could promote the formation of the reactive oxygen species, such as superoxide radical anion, peroxide and hydroxyl radicals, and hydrogen peroxide, the cytostatic activity of these substances is well known. These radical and radical-ion particles cause oxidative destructive reactions in cells, in other words, antimicrobial effect [20,21].

Along with the bactericide activity, due to the specific geometry and electronic structure, the complexes of metals with porphyrins have a significant effect on the crystallization and segmental orientation of macromolecules [22]. The pristine porphyrin molecules as well as their metal complexes are amphiphilic [23] or even hydrophobic [24] that promote their aggregation in the form of nanoparticles [23,25,26,27]. Depending on the nature of the metal included in metalloporphyrins, they exhibit a variable tendency to aggregation [28] that determines their ability to act as nucleating agents during polymer crystallization. In addition, porphyrins and their metal complexes have several binding sites, which facilitate the appearance of coordination interactions with the adjacent molecules [29]. Metal cations with chloride extra-ligands contained in the structure of porphyrin metal complexes can exchange ligands for polar fragments of the environment (for example, polymeric), such as oxygen-containing hydroxy- or carboxy-entities.

**Figure 1:**
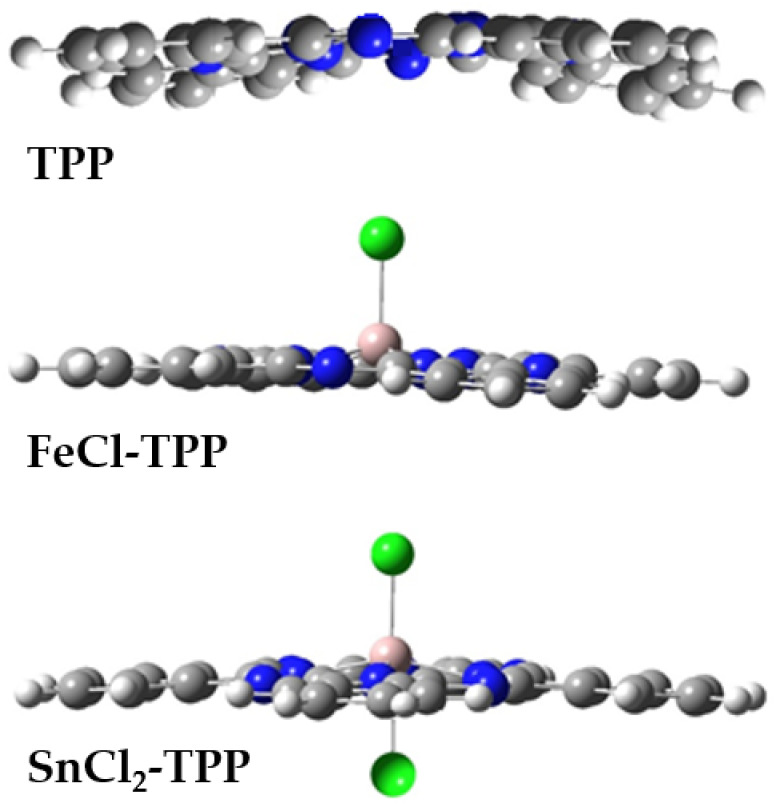
Side view on TPP, FeCl-TPP, and SnCl_2_-TPP.

Complexes of TPP with Sn(IV) (SnCl_2_-TPP) and Fe(III) (FeCl-TPP) are the most promising PHB dopants for creating polymeric materials for medical use. Sn-porphyrin metal-complexes are currently employed in the design of photocatalysts that promote the destruction of organic toxicants [29] and photosensitizers for medical diagnostics and therapy [30]. A significant advantage of the complexes of porphyrins with Sn (IV) is the presence of two extra ligands located on opposite sides of the plane of the porphyrin macrocycle (Figure 1). This structural peculiarity is responsible for the almost complete disability of SnCl_2_-TPP to aggregate in comparison with the complexes of porphyrins and metals in the oxidation states +2 and +3. FeCl-TPP molecules could have a strong interaction with PHB macromolecules; however, the presence of only one Cl as a ligand on only one side of the cycle leads to the potential ability of molecular aggregation.

Summarizing the above-mentioned topics, the goal of this study is the comparative analysis of the effect of TPP and its complexes with Sn (IV) and Fe (III) on the structural and dynamic parameters of a fibrous material based on PHB, as well as the detection of external factors’ impacts (ozonolysis, hydrolysis, annealing at 140 °C, etc.) on the structural and dynamic characteristics of the studied biodegradable polymer-porphyrin compositions.

## 2. Materials and Methods

### 2.1. Materials

For ES we used microbiologically synthesized PHB Series 16F Biomer (Schwalbach am Taunus, Germany) with a viscosity-average molecular mass (M_ν_) 2.06 × 10^5^ D, density ρ = 1.248 g/cm^3^, melting point Tm = 177 °C, initial degree of crystallinity ~63%.

TPP, FeCl_3_-TPP complex and SnCl_2_-TPP complex were obtained according to the procedure described earlier [31,32]. Their structural formulas are exhibited in Figure 1. The TPP complexes of Fe and Sn are readily soluble in organochlorine solvents, such as chloroform (CHCl_3_), but insoluble in water. To obtain the solution prepared for ES formation, the porphyrin dopants were dissolved in CHCl_3_ and then added to solutions of PHB in CHCl_3_. Thus, the both components have the same cosolvent.

The ES-formation solutions of PHB, PHB/TPP, PHB/FeCl_3_-TPP, and PHB/SnCl_2_-TPP in CHCl_3_ were prepared at a temperature of 60 °C by stirring on an automatic magnetic stirrer to complete uniformity. The concentration of PHB in the solution was 7 wt.%, the content of TPP, FeCl_3_-TPP, SnCl2-TPP was equal to 1, 3, and 5 wt.% relatively to the mass of PHB.

Fibers were obtained by ES on a single capillary laboratory setup with the following parameters: capillary diameter—0.1 mm, voltage—12 kV, distance between electrodes—18 cm, solution conductivity—10 μS/cm as described in [32] The fibers in the form of the mats were annealed at 140 °C ± 1 °C in the vacuum oven within the time interval 30–240 min. Right after then fibers were rapidly cooled to the room temperature.

### 2.2. Methods

X-ray structural analysis (XRD) of the samples was carried out by transmission recording. High-resolution two-dimensional scattering patterns were obtained using the S3-Micropix small- and wide-angle X-ray scattering system (CuKα radiation, λ = 1.542 Å) (Hecus X-ray Systems GmbH (Graz, Austria)). A Pilatus 100K (DECTRIS Ltd. (Baden, Switzerland)) detector was used, as well as a PSD 50M linear devise for the argon flow, a high voltage of 50 kV and a current of 1 mA on the Xenocs Genix (Xenocs SAS (Grenoble, France)) source tube. Fox 3D X-ray optics (Xenocs SAS (Grenoble, France)) were used to form the X-ray beam; the diameters of the forming slits in the collimator were 0.1 and 0.2 mm. To eliminate X-ray scattering in air, the X-ray mirror unit and the camera during signal accumulation were placed under the vacuum of (2–3) × 10^−2^ torr. The signal accumulation time was varied in the range 600–5000 s.

X-band electron paramagnetic resonance (EPR) spectra were recorded on an EPR-V automatic spectrometer (N.N. Semenov Federal Research Center for Chemical Physics, Russian Academy of Sciences, Moscow, Russia). To avoid saturation effects, the microwave power did not exceed 1 mW. The modulation amplitude was always significantly less than the resonance line width and did not exceed 0.5 G. A stable nitroxide radical TEMPO was used as a spin probe. The radical was incorporated into the fibers from the gas phase at a temperature of 50 °C for an hour. The concentration of the radical in the polymer was determined by double integration of the EPR spectra. As the reference was vacuum degassed TEMPO solution in CCl_4_ with a radical concentration of around 1 × 10^−3^ mol/L.

Simulation of EPR spectra was performed using the computer program described in This program is the modified version of the program described in [33]. The simulation was performed using nonlinear least-square algorithm. The values of the parameters under consideration were selected in such a way as to minimize the sum of squared deviations between the calculated spectra and the experimental ones. The initial program [33] allows calculating ESR spectra in the framework of Brownian rotational diffusion. The modified program [34] also allows taking into account the lognormal distribution of the rotational correlation times of paramagnetic molecules and calculating the spectra under the assumption of simultaneous rotation and quasi-librations (high-frequency low-amplitude vibrations of molecules near the equilibrium position [35,36]) of the radicals. The spectra were simulated using the following principal values of the g-tensor and the tensor of hyperfine interaction of an unpaired electron with a ^14^N nucleus: g_xx_ = 2.0093, g_yy_ = 2.0063, g_zz_ = 2.0022, A_xx_ = 7.0 G, A_yy_ = 5.0 G, A_zz_ = 35.0 G. The value of A_zz_ was determined experimentally from the EPR spectrum of TEMPO in PHB recorded at the temperature of liquid nitrogen. The obtained value is close to the value of A_zz_ given in [37].

Hydrolysis of samples in aqueous medium was studied at 70 °C ± 1 °C. Before the introduction of the radical, the samples exposed to water were dried in the vacuum oven to constant weight for 100 hr.

The differential scanning calorimetry (DSC) study was accomplished on a NETZSCH DSC 204 F1 instrument (NETZSCH-Geratebau GmBH (Selb, Germany) in an Ar atmosphere with a heating rate of 10 K/min. The average statistical error in the measurement of thermal effects was ±3%. The enthalpy of melting was calculated using the NETZSCH Proteus program. Thermal analysis was conducted according to the standard procedure [38]. Peak separation was performed using the NETZSCH Peak Separation 2006.01 software.

The scanning electron microscopy (SEM) to obtain geometric parameters of the fibrous materials was performed by TM-3000 scanning electron microscope (Hitachi, Ltd. (Tokyo, Japan)) at an accelerating voltage of 20 kV. Au layer of 10–20 nm was deposited on the surface of a nonwoven fibrous material sample. The computation and measurements of PHB fibrillar diameters were achieved using the software tool Java-based ImageJ version 1.52a (National Institutes of Health, Bethesda, MD, USA) and OriginPro 2018 (Origin Lab Corporation, Northampton, MA, USA). The histograms of diameter distribution for the loaded fibers of PHB have been built on the base of statistic estimation for 100 fibrillar elements patterned at SEM microphotographs.

## 3. Results and Discussion

### 3.1. Effect of Porphyrin and Metal-Porphyrines Concentrations on PHB Fiber Morphology

**Figure 2:**
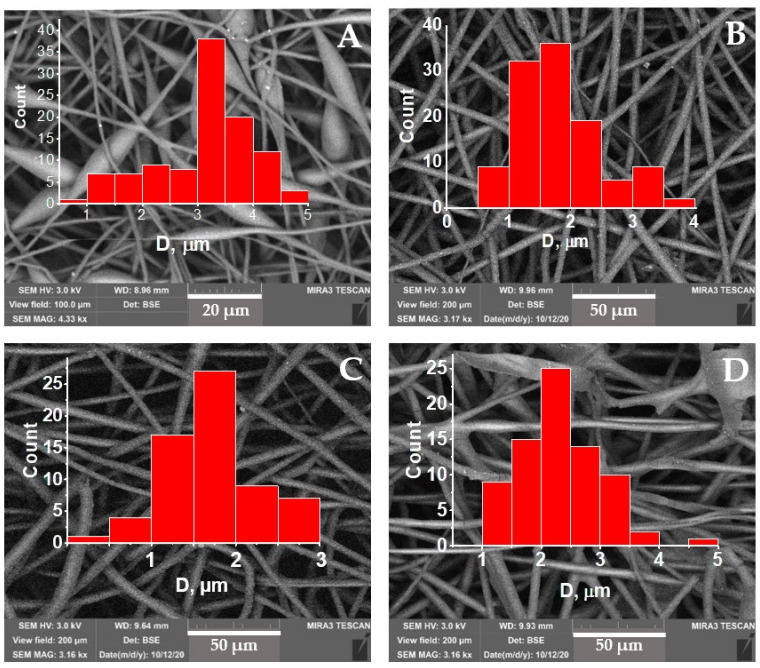
SEM images of the PHB fibrillar structures loaded by the metal-porhyrine complex of SnCl_2_. The concentration of SnCl_2_—PPT: (**A**) 0%, (**B**) 1%, (**C**) 3%, (**D**) 5%.

The pristine PHB fibers demonstrate the large concentration of ellipsoid structures as the anomalous bead-like fragments existing in the fibrous mats along with conventional cylindrical form being typical for electrospun fibers, see Figure 2A. As it was stated in the literature and a series of our works [39,40,41,42], the reason for the ellipsoids’ formation is low electrical conductivity (<1 μS/cm) and low surface tension of ES polymer solutions. The size of the ellipsoid beads in the transverse direction is 7.5 ± 2.5 µm, and in the longitudinal direction is ~17 ± 7 µm. The average diameter of the cylindrical fragments of the fiber is located in the range 1.5–4.5 µm. Comparative data on fiber diameter distribution is presented on inserts of Figure 2.

When the metal complexes of porphyrins were embedded into PHB fibers, the polymer morphology changed dramatically. At the SnCl_2_-TPP complex content 1–5 wt.%, the ellipsoid elements have completely disappeared; and for 1% of the complex, the fiber diameter distribution has the maximum at 1.75 ± 0.25 μm. The distribution asymmetry (insert in Figure 2B) manifested in the predominance of thin fibers’ fraction < 1.75 μm is likely a consequence of the jet splitting. With an increase in the concentration of FeCl-TPP from 3 to 5%, the fibers with a diameter of 3 μm prevail; see the corresponding histograms in Figure 2C,D.

The above observations can be explained as follows. When the metal-TPP complexes were added to the polymer electrospinning solution, ionic conductivity arises, which leads to the leveling of the surface tension of the solution drop and to the stability of the electrospinning process. The relative decrease in the fiber diameter is likely to be in correlation with the value of the electrical conductivity of the solution used for the polymer fibers formation. The bed-like entities disturbed the normal statistic distribution of the fibrils which caused to anomalous histogram. In this regard, we reconstructed the Figure 2A under condition that the anomalous beds should be excluded from statistical evaluation.

It is necessary to note that polydispersity with different locations of the distribution maximum in fiber diameters was observed for all compositions under study (see the corresponding histograms in Figure 2). Naturally, the polydispersity is due to the splitting effect in the primary polymer jet during electrospinning. The splitting effect can be associated with both an increase in the electrical conductivity of solution and a decrease in the surface tension at the polymer-air interface. Presumably, both effects could arise as a result of the metalloporphyrin introduction.

Thus, the introduction of TPP complexes leads to a change in the geometry and morphology of the fibers in the nonwoven fibrous material. The changes are undoubtedly associated with structural features of the PHB filament, which we will describe in the further sections by the methods of XRD, DSC, and EPR of the paramagnetic probe.

### 3.2. XRD Patterns of PHB Fibers Comprising the Porphyrines’ Complexes

The intermolecular interaction among the metal—porphyrin complex particles, as well as among these particles and PHB molecules, significantly depends on the nature of the complexes. During ES, the porphyrin molecules are attracted each other and, as a result, much larger aggregates are observed. The interaction of these particles with PHB macromolecules could be extremely small. In contrast with the pristine porphirin the FeCl-TPP complex has a strong interaction with PHB macromolecules. The presence of an extra-ligand chloride in the metal complex leads to intermolecular repulsion, i.e., the molecules FeCl-TPP are aggregated into smaller particles. In contrast, SnCl_2_-TPP complexes are statistically distributed in the polymer matrix due to the repulsive forces between a pair of Cl^−^ groups (see Figure 1). The interaction of SnCl_2_-TPP with PHB macromolecules is weaker than that of FeCl-TPP. This picture of intermolecular interactions determines the structural and dynamic characteristics of ultrathin fibers.

**Figure 3:**
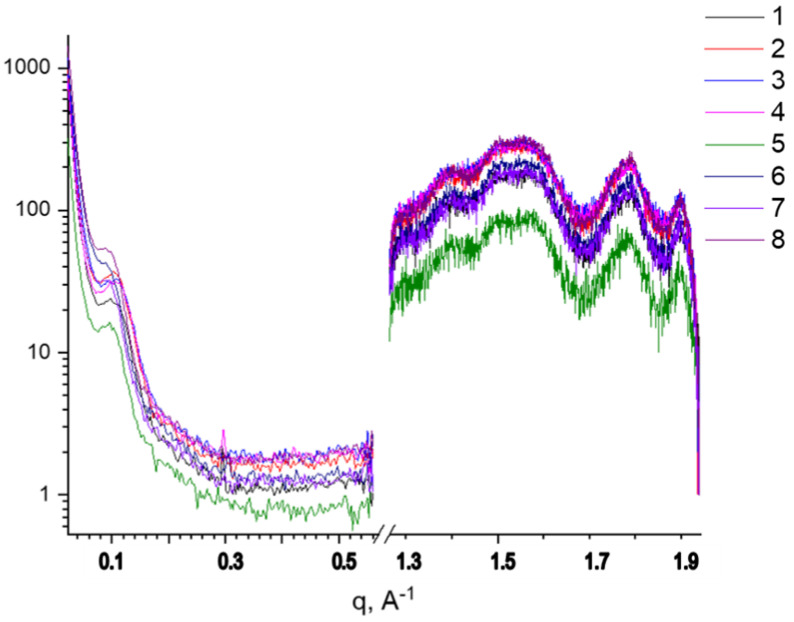
Diffractograms of PHB fibers containing metal-porphyrin complex (FeCl-TPP) before (1–4) and after annealing (5–8). PHB: nonannealed (1) and annealed (5); PHB-TPP-FeCl (1%): nonannealed (2) and annealed (6); PHB-TPP-FeCl (3%): nonannealed (3) and annealed (7); PHB-TPP-FeCl (5%): nonannealed (4) and annealed (8).

The study of initial PHB fibers and fibers from PHB loaded with TPP, SnCl_2_-TPP, and FeCl-TPP of various compositions just after ES and after annealing at 140 °C, was carried out by X-ray diffraction at wide and small angles. Figure 3 demonstrates the typical high-angle diffractograms of PHB/FeCl-TPP fibers.

The average effective crystallite size L_hkl_ in the crystallographic hkl direction was determined from the integral half-width of the line of the corresponding X-ray reflection using the Selyakov-Scherrer formula,

Δ_hkl_ (2θ) = λ/L_hkl_ cosθ_m_ (1)

The value of the large period was calculated by the formula,

d = nλ/2θ_m_ (2)

where d—long period, λ = 1.542 Å wavelength of CuKα—radiation, θ_m_—diffraction angle, and n—the order of reflection.

**Figure 4:**
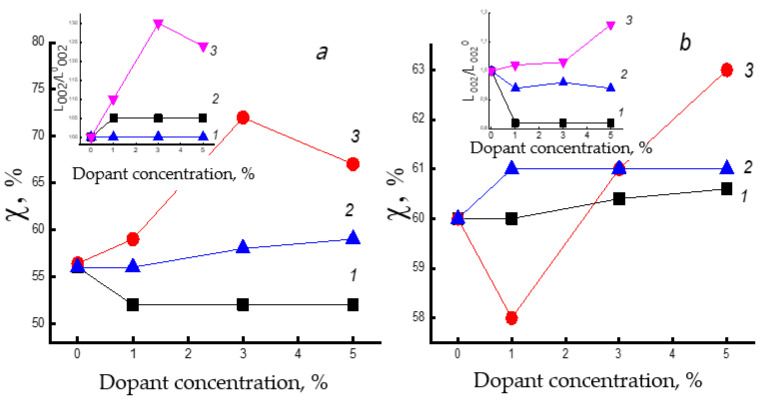
Crystallinity degree (χ) dependence and relative longitudinal size (L_00_2/L^0^_00_) evolution (the inserts) as functions of the dopant concentration. (**a**) initial fibers, (**b**) the same fibrous samples after annealing at 140 °C. 1—TPP, 2—SnCl_2_-TPP, 3—FeCl-TPP.

Figure 4a exhibits the dependences of the degree of crystallinity and the relative longitudinal size of crystallites on the concentration of the dopants. It is remarkably visible, that both parameters increase with the introduction of FeCl-TPP (the size of the crystallites increased by 30%). In PHB/TPP and PHB/(SnCl_2_-TPP) fibers, the changes in the degree of crystallinity and the longitudinal size of crystallites are within the experimental errors. The significant effect of FeCl-TPP on the crystal structure of the polymer is due to strong interaction of the additive molecules with the polymer macromolecules and the formation of dopant particles that can serve as the nuclei of crystallization. It should be noted that with an increase in the FeCl-TPP concentration from 3% to 5%, only a slight decrease in the degree of crystallinity and the longitudinal size of crystallites is observed. It takes place due to the aggregation of complexes into larger particles, which does not lead to a further increase in the proportion of crystallites during ES.

Thermal sterilization of polymeric materials remains the most common method in clinical practice. The recommended hot air annealing temperature required for the death of all microorganisms is 160 °C, however, the mode allowed for processing materials based on PHB fibers is limited to 140 °C, which is due to the onset of polymer degradation. Figure 4b exhibits the dependences of the degree of crystallinity and the longitudinal size of crystallites of the fibers annealed at 140 °C for 2 h on the concentration of the dopants. One can see that when 1% of FeCl-TPP is added, a decrement in the degree of PHB crystallinity is observed, and with an increment in the concentration of the metal complex, the degree of crystallinity increases sharply. The observed effects can be explained on the basics of knowledge, that crystallites and amorphous regions in a polymer generally do not correspond to the minimum free energy. The tendency of an amorphous-crystalline system to a minimum of free energy is facilitated by annealing the polymer, when macromolecules acquire sufficient mobility, while the crystallites tend to increase the longitudinal size. It seems that an increment in the degree of crystallinity can be expected upon annealing the sample. However, at FeCl-TPP concentration of 1%, the degree of crystallinity decreases. We explain the observed phenomenon as follows. At 140 °C, linear structures, edged surfaces, and defected crystal regions are thawed, while FeCl-TPP particles diffuse into such systems and fix in them due to the strong intermolecular interaction of this metal complex with PHB molecules. As a result, such areas are decompressed and do not give a signal in XRD. Annealing of PHB/TPP and PHB/(SnCl_2_-TPP) fibers at 140 °C leads to an insignificant increment in the degree of crystallinity.

### 3.3. Dynamic Characteristics of the Amorphous Phase of Ultrafine Fibers Loaded with Porphyrines

**Figure 5:**
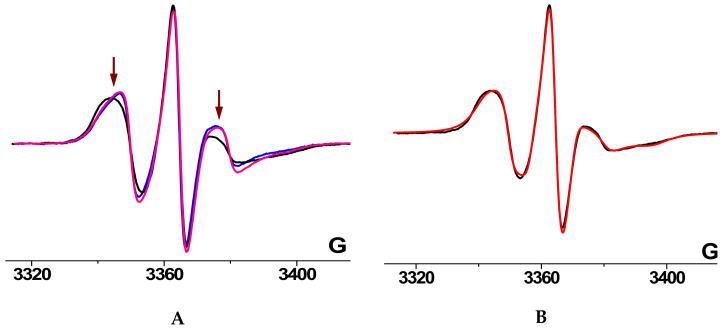
EPR spectra changes after annealing at 140 °C. (**A**) EPR spectra of TEMPO radical in PHB samples containing 5% TPP, before annealing (blue line) and after annealing for 90 (pink line) and 240 min (black line). The spectra are normalized to the intensity of the central component; the arrows indicate the regions of the greatest change in the line shape. (**B**) Spectrum simulation for the samples annealed for 240 min (black line—experimental spectrum, red line—simulation result).

The structure of the amorphous regions is largely determined by the degree of crystallinity of the polymer. As a result, the addition of porphyrin metal complexes to the PHB fiber changes not only the degree of PHB crystallinity but also the morphology and molecular dynamics in the amorphous regions. The molecular mobility of the amorphous regions of the polymer was studied by the spin probe technique using the stable nitroxide radical TEMPO. The typical EPR spectra of the radical in the polymer matrixes under consideration are shown in Figure 5.

The widely used method for determining the rotational parameters of spin probes in polymers is the computer simulation of the EPR spectra. It was found that for high-quality simulation of the spectra of the studied systems it is necessary to take into account the anisotropy of the rotational mobility of radicals, the continuous (lognormal) distribution of paramagnetic molecules over the rotational diffusion coefficients, as well as high-frequency low-amplitude vibrations of paramagnetic molecules near the equilibrium position—quasi-vibration [32,33,34,43]. As an illustration, Figure 5b shows the result of computer simulation of EPR spectrum of TEMPO in PHB containing 5% TPP after annealing at 140 °C for 240 min. Table 1 presents the parameters of the rotational mobility of the spin probes in the samples, the spectra of which are shown in Figure 5.

It was found that in all systems under study, paramagnetic molecules rotate anisotropically. The coefficients of the rotational diffusion of the radicals around X axis of g-tensor (N−O bond) do not exceed 2 × 10^6^ c^−1^, while the coefficients of rotational diffusion around Y and Z axes are in the range 10^7^–10^8^ c^−1^. Such anisotropy may indicate the interaction of probe molecules with functional groups of polymer molecules due to p-orbitals of nitrogen and oxygen atoms. A wide distribution of rotational mobility of the radicals was also revealed. This is caused by inhomogenity of the structure of the amorphous region of the polymers. Indeed, the amorphous phase is a set of structures characterized by different packing densities and different molecular dynamics of polymer chains. The EPR spectrum is a superposition of the spectra of radicals located in different regions of the amorphous phase and, therefore, having different mobility.

It should be noted that simulation of EPR spectra of low-molecular-weight dopants in polymers in the range of rotational correlation times of 10^−7^–10^−8^ s is a very time-consuming procedure. In addition, the present work is aimed at the qualitative analysis of changes in the radicals’ mobility as a result of treatment of the polymer matrix, and not at the interpretation of the exact values of the rotational parameters. In such a case it is useful to introduce a parameter that qualitatively characterizes the rotational mobility of radicals, can be determined without spectra simulation, and has sufficient sensitivity to small changes in the shape of the spectrum, such as the changes shown in Figure 5b. It can be seen that the greatest difference in the spectra is observed in the region of high-field and low-field components (indicated by arrows in the figure); therefore, as a parameter of the spectrum shape, we chose the value calculated as follows [44,45]:

τ_c_* = Δ*H*_+1_ × [(*I*_+1_/*I*_−1_)^0.5^ − 1] × 6.65 × 10^−10^ [s] (3)

Here Δ*H*_+1_ is peak-to-peak width of the low-field spectral component; *I*_+1_ and *I*_−1_ are intensities of the low-field and high-field components correspondently.

This formula was proposed for the determination of the rotational correlation times of the nitroxide radicals of the piperidine series in the case of their isotropic rotation in the range of rotational correlation times of 5 × 10^−11^ s ≤ τ_c_* ≤ 1 × 10^−9^ s. In this region, the EPR spectrum consists of three well-resolved components, the width of which is described within the framework of the Redfield theory [46]. In our case, this parameter qualitatively characterizes the shape of the EPR spectrum and can be considered as a characteristic correlation time. The values of τ_c_* for the three above systems are shown in Table 1. It can be seen that this value reflects the deceleration of the rotation of radicals in the PHB/TPP system (5%) upon sample annealing.

**Figure 6:**
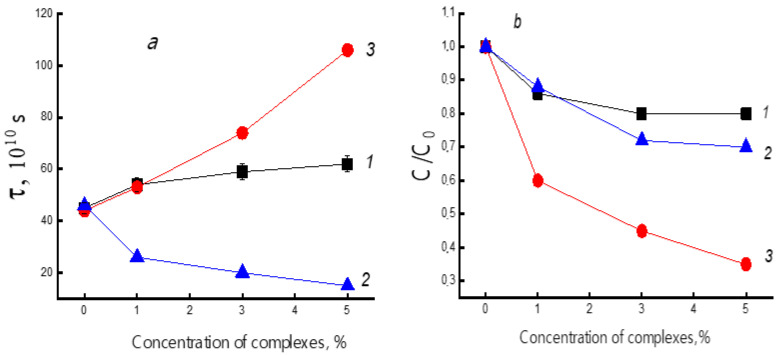
Dependences of probe correlation time τ (**a**) and relative probe concentration C/C_0_ (**b**) on dopant content: TPP (1), SnCl_2_—TPP (2), and FeCl-TPP (3).

Figure 6a shows the change in the rotational correlation time of spin probes with an increase in the concentration of complexes. The highest growth of τ_c_* is in the PHB/FeCl-TPP system; in the PHB/TPP fibers, the correlation time increases not so significantly. In PHB/SnCl_2_-TPP fibers, a decrease in τ_c_* is observed, although the degree of crystallinity determined using DSC increases with an increase in the additive content.

As shown above, doping of PHB with PHB/FeCl-TPP causes an increase in the polymer crystallinity. This phenomenon is accompanied by increase in the correlation time, and, consequently, a slowdown in the molecular dynamics of macromolecules. In PHB/TPP, the effect of slowing mobility is weak. For the polymer doped with SnCl_2_-TPP, a decrease in the rotational correlation time of the probe is observed, that is, an increase in the mobility of the polymer chains. This phenomenon is caused by the loosening effect of this metal complex, which is distributed in the system at the molecular level, and, therefore, the number of individual particles per unit volume of the polymer, in this case, is maximal.

Figure 6b demonstrates the dependences of the radical concentration on the polymer composition. It is seen that an increase in the PHB content leads to a decrease in the concentration of the radical. The strongest changes were observed in the polymer doped with FeCl_3_-TPP due to the strongest change in the degree of crystallinity in these samples. It is known that impurity molecules do not diffuse into crystal structures; their concentration in dense amorphous regions is insignificant.

### 3.4. Effect of Thermal Treatment on the Structural and Dynamic Parameters of PHB Doped with Porphyrin and Metalloporphyrins

Of great scientific and practical interest is the study of the effect of sterilization on materials and products for medical use. The choice of optimal conditions for polymer processing, in view of the fact that the aggressive action of temperature, radiation, oxidizer (oxygen, ozone), along with the disinfection of the material and the destruction of pathogenic microorganisms, can lead to a significant deterioration in a structural hierarchy and hence in polymer properties.

#### 3.4.1. Annealing of Samples at 140 °C

During ES performance, as a result of cooling and solidification, the polymer structure of ultrathin fibers can be far enough from the state of thermodynamic equilibrium. The imperfection in the crystalline phase and biopolymer morphology is manifested in the insufficient orientation of the segments in the fiber, as well as in an atypically low degree of crystallinity. To facilitate the transition of the polymer structure to equilibrium state the thermal annealing is used. The temperature impact allows intensifying segmental mobility and transferring the system to a more thermodynamically equilibrium state. The enhancing in the crystalline phase and especially additional crystallization occurs with the participation of transient polymer molecules located in intercrystalline area. Therefore, one should expect a change in the dynamics of spin probe that reflects the segmental mobility of PHB molecules in the intercrystalline fields. When comparing the mobility of the probe in the initial and annealed polymer samples contained different dopants, an opportunity appears to determine the effect of the additives on the polymer stability and dynamics of the intercrystalline structure.

**Figure 7:**
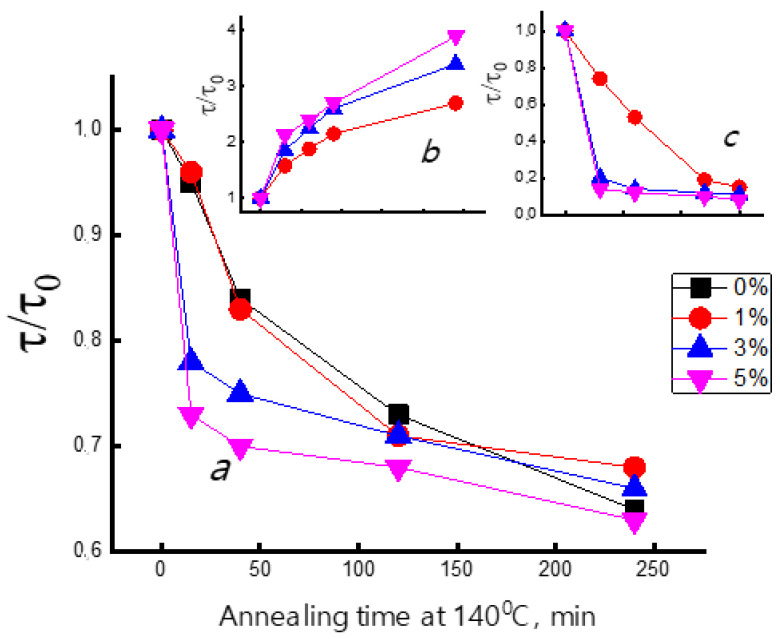
Dependence of the molecular dynamics expressed as relative correlation time (τ/τ_0_) from annealing time at 140 °C for the PHB electrospun fibers with (**a**) TPP, (**b**) SnCl_2_-TPP, (**c**) FeCl-TPP.

Figure 7 shows the dependencies of the characteristic rotational correlation time of spin probes on the annealing duration of the samples at 140 °C. It is seen that τ* decreases (rotational mobility of the radicals increases) significantly during annealing both for pristine PHB and PHB containing TPP, SnCl_2_-TPP, and FeCl-TPP additives. The observed dependences can be explained as follows. During the fibers’ fabrication, an essential proportion of the transitive macromolecules in transitive conformation are formed, but their segmental mobility is frozen as a result of the fiber jet solidification at room temperature. Annealing at 140 °C enhances macromolecular mobility that after gradual cooling leads to additional crystallization with the growth of crystallite sizes owing to the present of transitive straightened macromolecules. The spin probe molecules cannot penetrate into crystallites; therefore, their rotational mobility reflects the molecular mobility only in amorphous areas and in the areas of transitive macromolecules. After crystallization of the transitive areas the radicals are located predominantly in the amoprhous areas, so their average rotational mobility increases. Indeed, the absorption capacity of the fibers for the probe molecules decreases with an increase in the annealing time. For example, in the initial PHB fibers, the concentration of the radical was 0.75 × 10^15^ spin/cm^3^, while after annealing for 90 min, it decreased to 0.53 × 10^15^ spin/cm^3^, i.e., decreased 1.4 times. At the same conditions, the concentration of the radical in the PHB/SnCl_2_-TPP composition decreases 2.1-fold, and in the system PHB/FeCl-TPP—10-fold after annealing the samples with 5% additive for 2 h. The greatest decrease in content of the probe for the system PHB/FeCl-TPP corresponds to the greatest decrease in the rotational correlational time of the radicals.

#### 3.4.2. Exposure of Samples in Water at 70 °C

The structure and segmental dynamics of biodegradable materials for biomedicine significantly affect diffusion of drugs loaded into polymers and, consequently, the kinetic profiles of active component release. Since the polymer in the body functionalizes often in the aquatic environment, it is important to identify the changes in its structure and segmental mobility as a result of water action. Even a slight increase in the water content affects the diffusion processes of active agents in a polymer matrix. Previously, we studied the effect of an aqueous medium on the structure of PHB [47,48,49,50,51,52]. It was shown that under the influence of water, the PHB films degrade, the conformation of the polymeric chains altered, and the proportion of straightened segments decreases. It was necessary to establish the extent of these structural changes in the polymer compositions under investigation.

The exposure of fibrous materials to water medium in the present work was performed at 70° because at room temperature the saturation of polymer volume with water could last several days. When kept in water at 70 °C, the following processes occur simultaneously in the polymer:

(1) In accordance with the above description, heating the samples in water medium causes an increase in the degree of crystallinity and compaction of amorphous structures;
(2) Water molecules penetrating into the polymer matrix produce a plasticizing effect, as a result of which the degree of crystallinity also increases;
(3) When water molecules interact with PHB molecules and TPP or metalloporphyrin complexes, the hydrated complexes are formed, which loosen the structure of amorphous regions. After removal of boundless water, hydrated complexes could remain in the fiber;
(4) Water molecules penetrate into the accessible surfaces of crystallites and linear structures of amorphous areas, destroying these structures, as a result of which the degree of crystallinity decreases.

**Figure 8:**
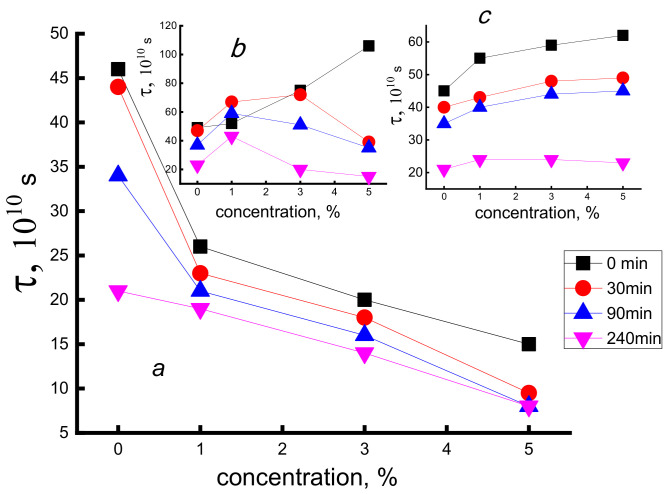
Dependence of time correlation of spin probe on the concentration of SnCl_2_-TPP (**a**), FeCl-TPP (**b**), and TPP (**c**) at a different times of water exposure at 70 °C.

The EPR probe was introduced into the fibers after their heating in an aqueous medium and subsequent drying in vacuum during 2 days until constant weight. The test results are shown in Figure 8. It is seen that in the samples containing various porphyrin complexes in diverse concentrations and exposed in water for different times, the intensity of the above processes is essentially changed. In PHB fibers loaded by TPP-SnCl_2_ (curves in Figure 8a), the mobility of spin probes increases (correlation time decreases) more than twice after maximal exposure (for 5 h). In the fibers with TPP completely different patterns of changes in molecular mobility are observed; at all concentrations of the additive and exposure time, the correlation time monotonically increases. In fibers containing FeCl-TPP, water exposure causes a complicated dependence of τ* on the additive concentration as the curves with maximum, see Figure 8b.

**Figure 9:**
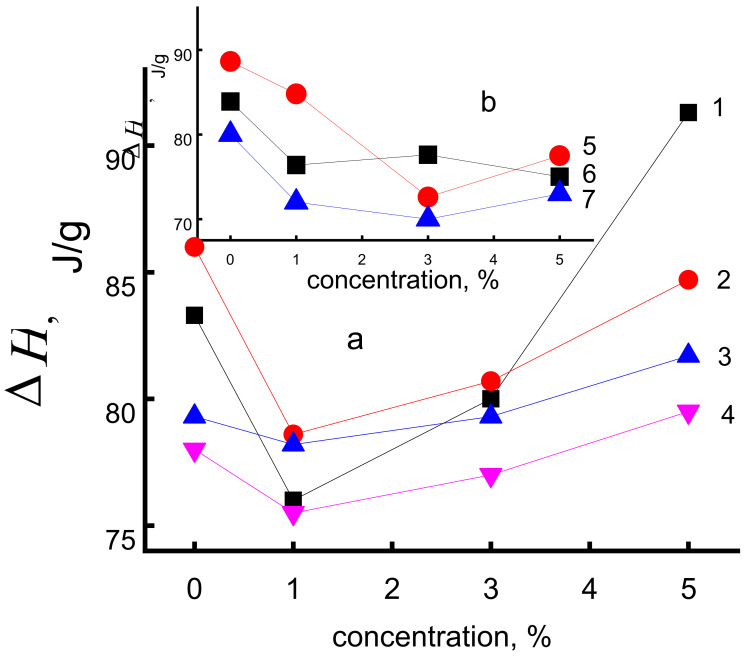
Dependence of melting enthalpy on the SnCl_2_-TPP (**a**) and TPP (**b**) content. Durability of water treatment (in min) at 70 °C: 1—0, 2—30, 3—120, 4—240, 5—0, 6—120, 7—240.

To compare the dopants impact on PHB structure the enthalpies of melting for crystalline fraction of PHB after water treatment are presented in Figure 9. In the case of SnCl_2_-TPP dopant (curves 1–4) at all times of annealing, there are clearly observed the extreme dependences of Δ*H* on wt.%. After the minimum point at 1 wt.%, the further growth in the enthalpies is observed that means the increasing of the crystallinity of the polymer and is in good agreement with time correlation dependence in Figure 8a. In the case of TPP dopant the extremum in enthalpy is manifested not so clearly (curves 5–7). On the basis of comparing of Figure 8c and Figure 9b we can suppose that treatment of PHB-TPP polymer in water at 70 °C causes an increase in the proportion of straightened polymer chains.

In general, the results obtained indicate that the doping of PHB with TPP and its metallo-complexes has a significant and often multidirectional effect on structural changes in the polymer during its processing for the purpose of sterilization. This observation opens up the possibility of manufacturing materials from PHB with predetermined properties.

## 4. Conclusions

Generally, it can be concluded that doping of PHB with TPP and complexes TPP with Sn(IV) and Fe(III) leads to significant change in the geometry and morphology of the polymer fibers fabricated using ES method. According to the dynamic and structural exploring the annealing of ultrathin PHB fibers as well as their exposure in aqueous medium strongly affects the structure of the amorphous and crystalline regions of the polymer, moreover, the changing of the structure depends on the nature and amount of the additives. The data obtained should be taken into account when developing a sterilization regime for medical devices made of PHB.

## Figures and Tables

**Table 1:** Parameters of the rotational mobility of TEMPO radicals in PHB containing 5% TPP at different times of sample annealing. σ is the width of the lognormal distribution of paramagnetic molecules with respect to rotational mobility; L—amplitudes of quasi-vibrations.

	0 min	90 min	240 min
D_x_ (s^−1^)/τ_c,x_ (s)	<2 × 10^6^/>8 × 10^−8^	<2 × 10^6^/>8 × 10^−8^	<2 × 10^6^/>8 × 10^−8^
D_y_ (s^−1^) (±0.1 × 10^7^)/τ_c,y_ (s)	6.4 × 10^7^/2.6 × 10^−9^	6.0 × 10^7^/2.8 × 10^−9^	5.5 × 10^7^/3.0 × 10^−9^
D_z_ (s^−1^) (±0.1 × 10^7^)/τ_c,z_ (s)	5.8 × 10^7^/2.9 × 10^−9^	5.7 × 10^7^/2.9 × 10^−9^	2.7 × 10^7^/6.2 × 10^−9^
σ_y_ (±0.05)	0.61	0.76	0.55
σ_z_*	2.50	2.50	2.50
L_y_ (±2°)	45°	46°	41°
L_z_ (±2°)	54°	55°	60°
τ_c_*	2.4 × 10^−9^	2.3 × 10^−9^	5.6 × 10^−9^

* The accuracy of determining the width of the distribution of radicals by rotational mobility around the molecular Z axis is low; in the resulting attempt at computer modeling, this value did not vary.

## Data Availability

Not applicable.

## References

[1] Raturi G., Shree S., Sharma A., Panesar P.S., Goswami S. (2021). Recent, approaches for enhanced production of microbial polyhydroxybutyrate: Preparation of biocomposites and applications. Int. J. Biol. Macromol..

[2] Briassoulis D., Athanasoulia P.T.-G. (2021). Alternative optimization routes for improving the performance of poly(3-hydroxybutyrate) (PHB) based plastics. J. Clean. Prod..

[3] Sirohi R., Pandey J.P., Gaur V.K., Gnansounou E., Sindhu R. (2020). Critical overview of biomass feedstocks as sustainable substrates for the production of PHB. Bioresour. Technol..

[4] Zhao X.-H., Niu Y.-N., Mi C.-H., Gong H.-L., Yang X.-Y., Cheng J.-S.-Y., Zhou Z.-Q., Liu J.-X., Peng X.-L., Wei D.-X. (2021). Electrospinning nanofibers of microbial polyhydroxyalkanoates for applications in medical tissue engineering. J. Polym. Sci..

[5] Chou S.-F., Carson D., Woodrow K.A. (2015). Current strategies for sustaining drug release from electrospun nanofibers. J. Control. Release.

[6] Xie J., Shen H., Yuan G., Lin K., Su J. (2021). The effects of alignment and diameter of electrospun fibers on the cellular behaviors and osteogenesis of BMSCs. Mater. Sci. Eng..

[7] Karpova S.G., Ol’khov A.A., Shilkina N.G., Kucherenko E.L., Iordanskii A.L. (2017). Influence of Drug on the Structure and Segmental Mobility of Poly(3-Hydroxybutyrate) Ultrafine Fibers. Polym. Sci. Ser. A.

[8] Karpova S.G., Lomakin S.M., Popov A.A., Ol’khov A.A., Iordanskii A.L., Shilkina N.S., Gumargalieva K.Z., Berlin A.A. (2016). Nonwoven blend composites based on poly(3-hydroxybutyrate)–chitosan ultrathin fibers prepared via electrospinning. Polym. Sci. Ser. A.

[9] Olkhov A.A., Staroverova O.V., Bonartsev A.P., Zharkova I.I., Sklyanchuk E.D., Iordanskii A.L., Rogovina S.Z., Berlin A.A., Ishchenko A.A. (2015). Structure and properties of ultrathin poly-(3-hydroxybutirate) fibers modified by silicon and titanium dioxide particles. Polym. Sci. Ser. D.

[10] Karpova S.G., Olkhov A.A., Bakirov A.V., Chvalun S.N., Shilkina N.G., Popov A.A. (2018). Poly(3-hydroxybutyrate) Matrices Modified with Iron(III) Complexes with Tetraphenylporphyrin. Analysis of the Structural Dynamic Parameters. Russ. J. Phys. Chem. B.

[11] Karpova S.G., Ol’khov A.A., Krivandin A.V., Shatalova O.V., Lobanov A.V., Popov A.A., Iordanskii A.L. (2019). Effect of Zinc–Porphyrin Complex on the Structure and Properties of Poly(3-hydroxybutyrate) Ultrathin Fibers. Polym. Sci. Ser. A.

[12] Karpova S.G., Ol’khov A.A., Lobanov A.V., Popov A.A., Iordanskii A.L. (2019). Biodegradable compositions of ultrathin poly-3-hydroxybutyrate fibers with MnCl–tetraphenylporphyrin complexes. Dynamics, structure and properties. Nano-Technol. Russ..

[13] Kotresh T.M., Ramani R., Jana N., Minu S., Shekar R.I., Ramachandran R. (2021). Supermolecular Structure, Free Volume, and Glass Transition of Needleless Electrospun Polymer Nanofibers. ACS Appl. Polym. Mater..

[14] Sharma S.K., Pujari P.K. (2017). Role of free volume characteristics of polymer matrix in bulk physical properties of polymer nanocomposites: A review of positron annihilation lifetime studies. Prog. Polym. Sci..

[15] Kazsoki A., Szabó P., Domján A., Balázs A., Bozó T., Kellermayer M., Farkas A., Balogh-Weiser D., Pinke B., Darcsi A. (2018). Microstructural distinction of electrospun nanofibrous drug delivery systems formulated with different excipients. Mol. Pharm..

[16] Papp J., Szente V., Süvegh K., Zelkó R. (2010). Correlation between the free volume and the metoprolol tartrate release of Metolose patches. J. Pharm. Biomed. Anal..

[17] Švajdlenková H., Šauša O., Adichtchev S.V., Surovtsev N.V., Novikov V.N., Bartoš J. (2021). On the Mutual Relationships between Molecular Probe Mobility and Free Volume and Polymer Dynamics in Organic Glass Formers: Cis-1,4-poly(isoprene). Polymers.

[18] Wei M., Wan J., Hu Z., Peng Z., Wang B., Wang H. (2017). Preparation, characterization and visible-light-driven photocatalytic activity of a novel Fe(III) porphyrin-sensitized TiO_2_ nanotube photocatalyst. Appl. Surf. Sci. Part B.

[19] Bayat F., Karimi A.R., Adimi T. (2020). Design of nanostructure chitosan hydrogels for carrying zinc phthalocyanine as a photosensitizer and difloxacin as an antibacterial agent. Int. J. Biol. Macromol..

[20] Sousa J.F.M., Pina J., Gomes C., Dias L.D., Pereira M.M., Murtinho D., Dias P., Azevedo J., Mendes A., de Melo J.S.S. (2021). Transport and photophysical studies on porphyrin-containing sulfonated poly(etheretherketone) composite membranes. Mater. Today Commun..

[21] Lobanov A.V., Kholujskaya S.N., Komissarov G.G. (2004). The H_2_O_2_ as donor of electrons in catalytic reduction of inorganic carbon. Russ. J. Phys. Chem. B.

[22] Szuwarzynski M., Wolski K., Krukc T., Zapotoczny S. (2021). Macromolecular strategies for transporting electrons and excitation energy in ordered polymer layers. Prog. Polym. Sci..

[23] Gradova M.A., Zhdanova K.A., Bragina N.A., Lobanov A.V., Mel’nikov M.Y. (2015). Aggregation state of amphiphilic cationic tetraphenylporphyrin derivatives in aqueous microheterogeneous systems. Russ. Chem. Bull..

[24] Rabiee N., Yaraki M.T., Garakani S.M., Ahmadi S., Lajevardi A., Bagherzadeh M., Rabiee M., Tayebi L., Tahriri M., Hamblin M.R. (2020). Recent advances in porphyrin-based nanocomposites for effective targeted imaging and therapy. Biomaterials.

[25] Zheng F., Zhang Y., Han Y., Zhang L., Bouyssiere B., Shi Q. (2021). Aggregation of petroporphyrins and fragmentation of porphyrin ions: Characterized by TIMS-TOF MS and FT-ICR MS. Fuel.

[26] Lobanov A.V., Gromova G.A., Gorbunova Y.G., Tsivadze A.Y. (2014). Supramolecular associates of double-decker lanthanide phthalocyanines with macromolecular structures and nanoparticles as the basis of biosensor devices. Prot. Met. Phys. Chem. Surf..

[27] Zhang X., Wasson M.C., Shayan M., Berdichevsky E.K., Ricardo-Noordberg J., Singh Z., Papazyan E.K., Castro A.J., Marino P., Ajoyan Z. (2021). A historical perspective on porphyrin-based metal–organic frameworks and their applications. Coord. Chem. Rev..

[28] Faustova M., Mollaev M., Zhunina O., Nikolskaya E., Lobanov A., Shvets V., Yabbarov N. (2018). Cytotoxic activity evaluation of metalloporphyrins in binary catalyst system. FEBS Open Bio..

[29] Yoo H.-Y., Yan S., Ra J.W., Jeon D., Goh B., Kim T.-Y., Mackeyev Y., Ahn Y.-Y., Kim H.-J., Wilson L.J. (2016). Tin porphyrin immobilization significantly enhances visible-light-photosensitized degradation of Microcystins: Mechanistic implications. Appl. Catal. B Environ..

[30] Arnold D.P., Blok J. (2004). The coordination chemistry of tin porphyrin complexes. Coord. Chem. Rev..

[31] Nakagaki S., Machado G.S., Stival J.F., dos Santos E.H., Silva G.M., Wypych F. (2021). Natural and synthetic layered hydroxide salts (LHS): Recent advances and application perspectives emphasizing catalysis. Prog. Solid State Chem..

[32] Van S.P., Birrell G.B., Griffith O.H. (1974). Rapid anisotropic motion of spin labels. models for motion averaging of the ESR parameters. J. Magn. Reson..

[33] Budil D.E., Sanghyuk L., Saxena S., Freed J.H. (1996). Nonlinear-least-squares analysis of slow-motion EPR spectra in one and two dimensions using a modified Levenberg–Marquardt algorithm. J. Magn. Reson. Ser. A.

[34] Chernova D.A., Vorobiev A.K. (2011). Molecular mobility of nitroxide spin probes in glassy polymers: Models of the complex motion of spin probes. J. Appl. Polym. Sci..

[35] Isaev N.P., Kulik L.V., Kirilyuk I.A., Reznikov V.A., Grigor’ev I.A., Dzuba S.A. (2010). Fast stochastic librations and slow small-angle rotations of molecules in glasses observed on nitroxide spin probes by stimulated electron spin echo spectroscopy. J. Non-Cryst. Solids.

[36] Karpova S.G., Olkhov A.A., Popov A.A., Iordanskii A.L., Shilkina N.G. (2021). Characteristics of the Parameters of Superfine Fibers of Poly(3-hydroxybutyrate) Modified with Tetraphenylporphyrin. Inorg. Mater. Appl. Res..

[37] Karpova S.G., Ol’khov A.A., Tyubaeva P.M., Shilkina N.G., Popov A.A., Iordanskii A.L. (2019). Composite Ultrathin Fibers of Poly-3-hydroxybutyrate and a Zinc Porphyrin: Structure and Properties. Russ. J. Phys. Chem. B.

[38] Castellón E., Günther H., Mehling. S., Hiebler L., Cabeza F. (2008). Determination of the enthalpy of PCM as a function of temperature using a heat-flux DSC—A study of different measurement procedures and their accuracy. Energy Res..

[39] Olkhov A.A., Staroverova O.V., Gol’dshtrakh M.A., Khvatov A.V., Gumargalieva K.Z., Iordanskii A.L. (2016). Electrospinning of biodegradable poly-3-hydroxybutyrate. Effect of the characteristics of the polymer solution. Russ. J. Phys. Chem. B.

[40] Filatov Y.N., Filatov I.Y., Smul’skaya M.A. (2017). Role of macromolecular factor in polymer solution for electrospinning process. Fibre Chem..

[41] Shepa I., Mudra E., Dusza J. (2021). Electrospinning through the prism of time. Mater. Today Chem..

[42] Rodríguez-Tobías H., Morales G., Grande D. (2019). Comprehensive review on electrospinning techniques as versatile approaches toward antimicrobial biopolymeric composite fibers. Mater. Sci. Eng..

[43] Saalmueller J.W., Long H.W., Volkmer T., Wiesner U., Maresch G.G., Spiess H.W. (1996). Characterization of the motion of spin probes and spin labels in amorphous polymers with two-dimensional field-step ELDOR. J. Polym. Sci. Part B Polym. Phys..

[44] Buchachenko A.L., Wasserman A.M. (1973). Stable Radicals.

[45] Karpova S.G., Ol’khov A.A., Chvalun S.N., Tyubaeva P.M., Popov A.A., Iordanskii A.L. (2019). Comparative Structural Dynamic Analysis of Ultrathin Fibers of Poly-(3-hydroxybutyrate) Modified by Tetraphenyl–Porphyrin Complexes with the Metals Fe, Mn, and Zn. Nanotechnol. Russ..

[46] Redfield A.G. (1966). The theory of relaxation processes. Adv. Magn. Reson..

[47] Razumovskii L.P., Lordanskii A.L., Zaikov G.E., Zagreba E.D., McNeill I.C. (1994). Sorption and diffusion of water and organic solvents in poly(l -hydroxybutyrate) films. Polym. Degrad. Stab..

[48] Iordanskii A.L., Kamaev P.P., Hänggi U.J. (2000). Modification via preparation for poly(3-hydroxybutyrate) films: Water-transport phenomena and sorption. J. Appl. Polym. Sci..

[49] Kamaev P.P., Aliev I.I., Iordanskii A.L., Wasserman A.M. (2001). Molecular dynamics of the spin probes in dry and wet poly(3-hydroxybutyrate) films with different morphology. Polymer.

[50] Pankova Y.N., Shchegolikhin A.N., Iordanskii A.L., Zhulkina A.L., Olkhov A.A., Zaikov G.E. (2010). The characterization of novel biodegradable blends based on polyhydroxybutyrate: The role of water transport. J. Mol. Liq..

[51] Ventura H., Claramunt J., Rodríguez-Perez M.A., Ardanuy M. (2017). Effects of hydrothermal aging on the water uptake and tensile properties of PHB/flax fabric biocomposites. Polym. Degrad. Stab..

[52] Sangroniz A., Sarasua J.R., Iriarte M., Etxeberria A. (2019). Survey on transport properties of vapours and liquids on biodegradable polymers. Eur. Polym. J..

